# Why are we not evaluating multiple competing hypotheses in ecology and evolution?

**DOI:** 10.1098/rsos.160756

**Published:** 2017-01-11

**Authors:** Gustavo S. Betini, Tal Avgar, John M. Fryxell

**Affiliations:** 1 Department of Integrative Biology, University of Guelph, Guelph, Ontario, CanadaN1G 2W1; 2 Department of Biological Sciences, University of Alberta, Alberta, Edmonton, Canada T6G 2E9

**Keywords:** Platt, Chamberlin, strong inference, model selection, scientific method

## Abstract

The use of multiple working hypotheses to gain strong inference is widely promoted as a means to enhance the effectiveness of scientific investigation. Only 21 of 100 randomly selected studies from the ecological and evolutionary literature tested more than one hypothesis and only eight tested more than two hypotheses. The surprising rarity of application of multiple working hypotheses suggests that this gap between theory and practice might reflect some fundamental issues. Here, we identify several intellectual and practical barriers that discourage us from using multiple hypotheses in our scientific investigation. While scientists have developed a number of ways to avoid biases, such as the use of double-blind controls, we suspect that few scientists are fully aware of the potential influence of cognitive bias on their decisions and they have not yet adopted many techniques available to overcome intellectual and practical barriers in order to improve scientific investigation.

## 1. Introduction

**Figure 1.**
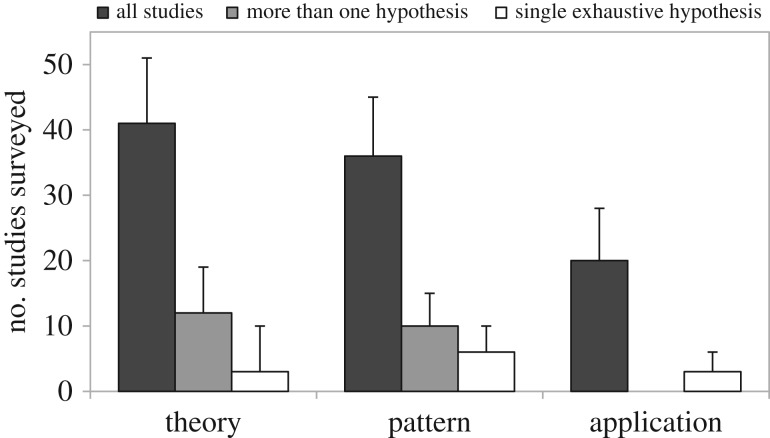
Number of studies identified in all survey as motivated by pattern, theory or application. Three studies were purely descriptive, lacking a hypothesis and were not included in the figure. The black bars indicate the number of studies within each category that tested more than one hypothesis. Bootstrapped 95% CI are provided in table 1.

In a much celebrated paper, Platt suggested that scientific progress is promoted by the consistent application of strong inference (box 1), i.e. devising and testing multiple hypotheses with crucial experiments and re-evaluating them to refine the possible explanations for a given phenomenon [1]. The method of multiple hypotheses was first developed by Chamberlin in 1890 [2], who believed that scientists usually ‘fall in love’ with their favourite hypothesis, leading to the unfortunate practice of trying to fit all evidence into a single explanation instead of finding genuine explanations for the phenomenon they study. The solution, according to Chamberlin, is to cultivate the habit of developing and comparing alternative hypotheses to explain any phenomenon observed. Platt pointed out that multiple hypothesis testing was responsible for the fast growth of molecular biology in the 1960s, demonstrating its utility for any field that seeks rapid scientific progress. Since then, multiple hypothesis testing has become a staple of graduate qualifying exams and is commonly extolled in methodological textbooks. Surprisingly, the method of multiple hypotheses is rarely used in ecology and evolution [3]. In a sample of the literature (figure 1 and table 1), we found that only 21 of a 100 randomly selected studies tested two hypotheses, and only eight tested more than two hypotheses (box 2). While descriptive studies are essential in ecology and evolution, because they can help us to generate hypotheses (one needs to know the pattern to be able to explain it), the rarity of multiple hypothesis testing raises an important question: if it is such a powerful tool for scientific investigation, why is multiple hypothesis testing used so sparingly by ecologists and evolutionary biologists?

Box 1. **The method of strong inference.**

Platt envisioned a scientific method, which consists of applying four steps in to every problem in science, ‘formally and explicitly and regularly:

(1) Devising alternative hypotheses;
(2) Devising a crucial experiment (or several of them), with alternative possible outcomes, each of which will, as nearly as possible, exclude one or more of the hypotheses;
(3) Carrying out the experiment so as to get a clean result;
(1')Recycling the procedure, making subhypotheses or sequential hypotheses to refine the possibilities that remain; and so on.’ [1, p. 347]

Platt gave special attention to the design of ‘alternatives and crucial experiments’ that would exclude scientific hypothesis, not simply the design of any experiment and outcome, or the ones that would corroborate a given hypothesis. Although Platt does not make this link, strong inference is based on the falsifiability of the philosopher Karl Popper. For Popper, inductive logic cannot be used to prove an idea because it is limited by one's own experience and there is always the possibility that a new experience will prove our ideas were wrong. Thus, it does not matter how many times we observe a phenomenon, we can never be sure that it will happen again in the future. By the same token, hypotheses, which are causal explanations for the phenomena we observe, can never be proved based on inductive logic. Hypotheses can only gain levels of confidence as they fail to be rejected by the evidence. In other words, hypotheses can only be corroborated by experience (i.e. data), never proved. Thus, for Popper, the essence of science is to test a hypothesis constantly with critical experiments so that we increase our level of confidence in that idea.

Box 2. **The use of multiple hypotheses in ecology and evolution.**

To investigate whether ecologists and evolutionary biologists apply multiple hypotheses in their studies, we conducted a sample of the literature. We randomly selected 20 papers from each of five leading journals in the fields of ecology and evolution: *Ecology, Ecology Letters, Molecular Ecology, Evolution* and *Global Change Biology*. To select a paper from each journal, we estimated the number of papers published by a given journal between 2001 and 2011 using the Web of Science. We sorted the papers by publication date and then divided this number by 20 and selected one paper from each interval to have an equally distributed sample within a period of 10 years. For example, if a given journal published 10 000 papers between 2001 and 2011, we first organized all the papers by publication date and then selected one out of every 500 papers. We have excluded review papers as well as purely theoretical or methodological studies. For each paper, we have noted the number of research hypotheses and whether these are competing (i.e. their predictions apply to the same class of pattern), mutually exclusive and/or exhaustive (i.e. there could be no alternatives but the null). Furthermore, we have noted the number of predictions affiliated with each hypothesis and whether the authors employed any type of model comparison. Finally, we classified each paper as motivated by theory (i.e. deductive) or pattern (i.e. inductive). We also included a third category, inquiries motivated by application, necessary owing to the prevalence of published scientific work aimed at offering practical solutions to a particular real-world problem.

Out of the 100 surveyed papers, 36 were classified as motivated by patterns, six of which tested a single exhaustive hypothesis (i.e. there could be no alternatives but the statistical null; figure 1). Out of the remaining 30 pattern-motivated studies, five lacked any research hypothesis, 15 tested one hypothesis (with an average of 2.1 predictions per hypothesis), five evaluated two hypotheses (with an average of 1.4 predictions per hypothesis) and five evaluated more than two (with an average of 1.4 predictions per hypothesis). Out of the 10 studies evaluating more than one hypothesis, only five employed some type of formal model comparison, whereas one tested mutually exclusive hypotheses.

We found that out of the 100 surveyed papers, 41 were classified as motivated by theory, three of which tested a single exhaustive hypothesis (figure 1). Out of the remaining 38 theory-motivated studies, four lacked any research hypothesis, 22 tested one hypothesis (with an average of 2.5 predictions per hypothesis), nine evaluated two hypotheses (with an average of 2.4 predictions per hypothesis) and three evaluated more than two (with an average of 2.7 predictions per hypothesis). Out of the 12 studies evaluating more than one hypothesis, only four employed some type of formal model comparison, but three tested mutually exclusive hypotheses.

Finally, 20 studies were classified as motivated by application, three of which tested a single exhaustive hypothesis (figure 1). Out the remaining 17, 15 lacked any research hypothesis and the other two tested a single hypothesis (with an average of 1.5 predictions per hypothesis). The remaining three papers in our survey focused on describing a pattern and thus lacked a hypothesis.

Surprisingly, inductive studies, motivated by patterns, showed a similar rate of multiple hypothesis usage compared with deductive studies, motivated by theory (28% and 27%, respectively), but a lower number of predictions per hypothesis (1.3 and 2.5 prediction per hypothesis, respectively) indicating that pattern-motivated study usually tested a single ‘prediction’.

**Table 1.** Number of studies and bootstrapped 95% CI calculated for theory-motivated studies (theory), pattern-motivated studies (pattern) and application-motivated studies (application). Bootstrapped 95% CIs were also provided for studies that tested more than one hypothesis (there were no papers that tested more than one hypothesis classified as application-motivated study). Code for bootstrapping is provided in the electronic supplementary material.

	theory	pattern	application
all studies	41 (31, 51)	36 (27, 46)	20 (12, 28)
more than one hypothesis	12 (7, 18)	10 (5, 15)	0
single exhaustive hypothesis	3 (0, 7)	6 (2, 10)	3 (0, 6)

We believe that the answer to this question stems from a number of intellectual and practical barriers. Here, we give our perspective on the subject and, supported by empirical data, argue that intellectual barriers arise from cognitive bias, the predictable tendency of humans to think somewhat irrationally [4]. We also identify several practical barriers, such as the elevated number of treatments and observational data required to test multiple hypotheses. We finish each section with a list of ways to overcome both intellectual and practical barriers. Our main thesis is that although the method of multiple hypotheses is difficult to implement and communicate, single hypothesis testing is prone to many problems arising from cognitive bias. Scientists have developed a number of ways to avoid such biases, such as the use of double-blind controls. We argue that the method of multiple hypotheses should also be considered a standard of sound scientific practice.

## 2. Barriers

In what follows, we try to understand the barriers that stop us from using multiple hypothesis testing more frequently in our research. We consider two distinct classes of barrier: intellectual and practical. Intellectual barriers are those that limit our ability or motivation to *consider* alternative explanations when approaching a scientific question. Practical barriers are those limiting our ability or motivation to *execute*, *analyse* and *publish* scientific investigations of multiple hypotheses. Here, we exclude the common argument that ecology and evolution are less prone to strong inference than molecular biology because of the need to rely on observational and comparative studies, which are not suitable for strong inference [5,6]. Although this seemingly attractive argument has generated much debate in our field [7,8], Chamberlin was a geologist and developed the method of multiple hypotheses with observational data in mind. Therefore, it is reasonable to suppose that Chamberlin's method should also be appropriate for other areas of scientific inquiry in which controlled experiments are not readily available. In fact, we argue that it is the often observational nature of ecological and evolutionary research that makes the use of multiple hypotheses so important, as means of considering all plausible processes that might have produced the observed pattern.

### 2.1. Intellectual barriers

Although we like to see ourselves as creatures of logic, making decisions that guide our lives based on rational steps, there is overwhelming evidence from experimental psychology demonstrating that humans are prone to many cognitive biases, which could accordingly limit the effectiveness of scientific decision-making [4,9]. Cognitive bias is an old acquaintance of scientists (e.g. experimenter's bias or the unconscious influence of the experimenter on the output of an experiment), and many methods have been developed to avoid such bias (e.g. double-blind controls). We nonetheless suspect that few scientists are fully aware of the potential influence of cognitive biases on our decisions. This is not surprising giving that the role of cognitive biases in science has been largely ignored (but see [10–12]).

In this section, we outline three cognitive biases that can interfere with our work: confirmation bias, pattern seeking and belief bias. Such biases work by limiting our ability to consider the possibility of alternative hypotheses, focusing our attention unnecessarily on a single or small set of favourite hypotheses that are constantly reinforced by unconscious processes. As a consequence, they limit our motivation to consider other potential explanations, because the evidence in favour of a single hypothesis becomes so overwhelming in the eyes of the scientist that it seems a complete waste of time to consider and test alternative ideas. This creates a positive feedback loop between the scientist and her/his favourite hypotheses that is hard to break, and sometimes hard to be understood by an outside viewer.

#### 2.1.1. Confirmation bias

Chamberlin was concerned with the tendency of some scientists to put more weight on evidence that supports favoured ideas more than other evidence that is available, which is today known as confirmation bias. He was not the first person to recognize the issue. The philosopher Francis Bacon had already pointed out that ‘the human understanding when it has once adopted an opinion (…) draws all things else to support and agree with it’, and he believed this was also true in science [13]. Although neither Chamberlin nor Bacon provided evidence for their argument, confirmation bias has been tested many times and can affect judgements that are expected to be based solely on logic [9,14]. In science, confirmation bias can be caused by the inclination of scientists to prefer some hypotheses over others or by the lack of alternative hypotheses in the field [11,15].

#### 2.1.2. Pattern seeking

Confirmation bias can be emphasized by the tendency of humans to find patterns [16]. Although pattern seeking is a useful and important component in our everyday behavioural repertoire, these benefits come at enormous cost: humans are well known to find patterns even where there are none to be found. Although we treat this as an error in cognition, there has been support for the idea that finding a non-existent pattern might be a selectively advantageous product of evolution, as long as the response provides a demonstrable fitness benefit [17]. This has important consequences for science, because results from experiments or observations are often not as clear as one might expect, and without a list of predictions from different hypotheses to guide the seeking process, our pattern-seeking mind tends to seize on any pattern available, even when dealing with random phenomena. Pattern seeking should be a concern in exploratory analysis and data mining, because finding a variable that is statistically significant just by chance is not as difficult as one might think [18]. For example, a regression analysis on two randomly generated independent variables will indicate a significant relationship 5% of the time. The higher the number of independent variables, the higher the possible combinations that will, just by chance, come out as significant. In fact, a combination of variable selection process (e.g. stepwise regression) with classical significance testing can provide low *p*-values and high coefficient of determination (*R*^2^), even when these variables are randomly generated [19]. These problems are even more evident when sample sizes are smaller, and studies are not replicated [20]. This is not to say that data mining is flawed in any way because it might serve as an important first step for generating hypotheses from complex ‘big’ datasets. However, data mining is not a valid route for evaluating scientific hypotheses.

#### 2.1.3. Belief bias

Logical arguments should be judged only by their internal consistency. However, sometimes logic and belief do not agree. For example, look at these two syllogisms

All mammals can walk.
Whales are mammals.
Therefore, whales can walk.
All flowers need water.
Roses need water.
Therefore, roses are flowers.

The first syllogism, despite being valid, is not believable (examples from [21]). The conclusion follows the premises, but one of the premises is false. In the second example, the premises are correct: we all know roses are flowers, and we all know flowers need water, but the syllogism is invalid (roses are flowers not because they need water). There have been many studies suggesting that when people face a conflict between logic and prior knowledge, they often respond on the basis of the latter, not the former. In science, belief bias could interfere with our evaluation of alternative hypotheses. If we are prone to decide on the validity of the logical structure of an alternative hypothesis based on our previous knowledge, we could judge hypotheses based on what our favourite hypothesis tells us about the world. Or it could be much simpler than that: when faced with results that do not confirm previous scientific knowledge, we might be inclined to reject the data themselves, on the assumption that something was done incorrectly; when the data are wrong, but tell us something that we were expecting, we tend to be less rigorous in our assessment of the evidence [12]. Empirical evidence suggests that researchers are more likely to find support for an effect that does not exist than to find evidence that could reject the effect [12,18]. This problem could be even more important because it is not clear how researchers determine whether a given piece of evidence is reliable or not [22] and one might place greater weight on the kinds of evidence that support their beliefs.

Taken together, these three cognitive biases, to which we are all susceptible, serve both as a reminder of why we should use multiple working hypotheses, and as a plausible explanation of why we are failing to do so.

Despite the fact that cognitive bias in science has been largely ignored, even among professional psychologists, there have been several cases where cognitive bias played an important role in the outcome of scientific investigations. One of the best-known examples is the study conducted by Mitroff about hypotheses associated with the moon by a group of Apollo scientists [23]. Mitroff started interviewing 42 high-level NASA research scientists three months before the first Apollo 11 landed on the moon, following up with similar interviews with the same group for the following three and a half years, during which Apollo missions 12–16 were conducted. Before the first Apollo mission, these scientists had published their conjectures about how the moon would look, which in some cases were quite different from each other. Mitroff found that, for a subgroup identified by their peers as the most attached to their theories, there was no change in their viewpoint even when confronted with overwhelming evidence to the contrary. Other well-known examples of cognitive bias in scientific programmes include (i) the case of Raymond Pearl, who insisted that the logistic equation was a general law applying to all populations, even when a large body of evidence proved otherwise [24]; (ii) the race to find the molecular structure of DNA [25] and (iii) the dispute over sociobiology [25]. We suggest that close examination of most scientific debates will find examples in which scientists fail to judge hypotheses solely on the evidence.

An active and exciting field in cognitive science explores the reasons for irrational decision-making. Some specialists blame this on heuristics, a set of rules for decision-making that worked at one point in our evolutionary history, but are inappropriate for the modern world [4]. Others question the very notion that logic is the basis for our decisions [26]. Regardless of which school of thought one favours, the important message for practising scientists is that we are all vulnerable to irrational conclusions. As physicist Richard Feynman once said, ‘The first principle is that you must not fool yourself--and you are the easiest person to fool’ [27]. We believe that the method of multiple hypotheses is extremely valuable in combating cognitive bias because it forces us to consider and collect evidence to support (or reject) a number of alternative explanations, not only a single hypothesis.

### 2.2. How can we overcome intellectual barriers?

Experimental psychologists have repeatedly demonstrated the commonality of cognitive bias in the form of optical illusions. Even when one is fully aware of such illusions, there is little one can do to avoid them based solely on our sensual perception. It seems that the best way to avoid cognitive bias is to ensure that objective forms of measurement are used to detect patterns. Fortunately, there are several ways one might minimize the effect of cognitive bias in science, so that one does not rely exclusively on one's perceptions.

#### 2.2.1. The null model

A null model generates a pattern in the absence of any biological process [28], forcing the researcher to think about many different hypotheses, which could potentially minimize the negative impacts of cognitive biases in science. Perhaps the best-known example of a biological null model is the Hardy–Weinberg equilibrium, predicting that gene frequencies will remain roughly constant over time in the absence of mate choice, mutation, selection, genetic drift, gene flow or meiotic drive. All these conditions are well known for causing changes in gene frequency and could each be seen as different hypotheses. The profound utility of the null Hardy–Weinberg equilibrium is that, if trait frequencies do exhibit change over time, then it must be because at least one of the six conditions has been violated. Other notable examples of productivity-enhancing null models include the ideal free distribution [29], predicting that in the presence of negative-density dependence but in the absence of limitations on movement or information, fitness should be equalized across a heterogeneous landscape, and the neutral theory of biodiversity [30], predicting structured community patterns in the absence of interspecies differences in competitive or dispersal ability. Hence, a well-defined null model is useful not only as an alternative hypothesis on its own, but also as a formal way of coming up with additional hypotheses, which are those relating to processes not represented in the null model.

It should be noted that biological models are very different from statistical null models, which may be less useful in avoiding cognitive biases. This is because a statistical null model, unlike a biological null model, is generated in opposition to a single hypothesis and therefore does not require alternative explanations for the biological phenomenon of interest. Moreover, although a statistical null model can also give rise to a null pattern that could be compared with observed patterns using frequentist inference approach (i.e. null hypothesis testing), subtle variation in biological hypotheses often requires more formal approaches to model competition based on information-theoretic basis, such as Akaike information criterion (AIC) or Bayesian information criterion (BIC). Using such methods, the degree of support for a given model (hypothesis) can be assessed relative to that for a more sophisticated array of alternative null models (e.g. the neutral theories of biodiversity or molecular evolution).

While often used as a tool for statistical inference, a set of regression models containing partially overlapping sets of explanatory variables does not necessarily represent a valid evaluation of multiple hypotheses unless each statistical model represents a unique research hypothesis [31]. In other words, model competition, based on AIC and BIC, is not a stepwise regression procedure. The candidate set of models tested in a model selection framework must be selected according to strict biological sense, as the results of model selection are only informative when considered in the context of the full set of alternatives [31,32]. This is, in part, because information-theoretic techniques will always rank the models that are being tested. If the models are not biological meaningful in the first place, how would one interpret the results of a given model competition?

Null models do not come easily. As biologists, we are trained to think of the underlying biological processes, not their absence. For example, take the Hardy–Weinberg equilibrium mentioned above. Evolutionary biologists want to understand how evolution happens, so they think about the mechanisms driving changes in allele frequencies, not about the mechanisms that might keep allele frequencies constant or situations under which these mechanisms are never at work. Hence, formulating an appropriate null model often requires a counterintuitive thought process. Nevertheless, given their enormous inferential importance, both ecologists and evolutionary biologists should always include an appropriate biological null model as part of our alternative hypothesis set.

#### 2.2.2. Creative thinking

It is far from simple to think outside of the existing box of theory and knowledge. Lack of creativity is a serious barrier to solving any problem, because we instinctively stick to traditionally used approaches and tools. Scientists are well-educated, informed and opinionated individuals. Ironically, it is our own prior knowledge that biases our way of approaching any new problem. Moreover, we often have a favourite theory or explanation, resulting in a confirmation bias. Creative thinking could help to avoid cognitive bias if used to consider processes that might result in the same observed pattern (what else, besides my favourite hypothesis, could be the mechanism that generated the observed pattern?). In some cases, one might even find that the overall scientific question is ill-posed [33]. Creative thinking can be taught and learnt [34], but we do not know of any example of creative thinking that helped to avoid cognitive bias. We suggest that we need to train ourselves to routinely apply tools of lateral and critical thinking as one possible way to avoid cognitive bias and promote the use of multiple alternative hypotheses as a standard part of our inquiry process.

#### 2.2.3. Work with the enemy

One useful antidote to perceptual bias is to work directly with other scientists with different perspectives. The results of such collaboration could yield a ‘more even-handed, informative, and constructive contribution to the literature than either side is likely or able to contribute on their own’ [35]. The debate among researchers with different points of view is crucial for the development of any scientific field, as argued by Hull [36], the philosopher of science. In ecology and evolution, many important debates helped to shape the fields as we know today, including the debate around the validity of the adaptationist programme, whether sociobiology could help understanding human behaviour, and the role of density dependence in population regulation [24,25,36,37]. However, it is also often the case that different scientific traditions have more in common than might be first assumed. As an example, prominent researchers with opposing points of view recently wrote a review on niche construction [35]. In so doing, they came to the important realization that their differences of opinion reflected a deeper dispute over the primacy of neo-Darwinism rather than a simple dispute about elements of niche theory. They also found that much of the disagreement was a result of ‘different usages of some key terms (e.g. evolutionary process)’. Deeper fundamental questions similarly underlay the sociobiology debate of the 1980s [25]. We propose that concerted collaboration between opposite sides of the same debate is the best way to evaluate the differences and similarities between opposite scientific traditions, which could help in the development of a given field.

#### 2.2.4. Blind analysis and crowdsourcing

Blind control has long been a staple of good experimental methodology in science. More recently, blind analysis has also become popular, at least in some fields [10,38,39]. The underlying concept is simple: the person or group analysing a dataset knows little about the experiment or observational data, so there is less chance that a given outcome will be favoured by the inherent bias of the investigator. This could be accomplished in many different ways. In the simplest case, one might ask a co-worker to change labels of treatments prior to analysis (e.g. A and B instead of high and low). However, blind analysis could also involve different teams of researchers applying their own statistical approaches to answer the question at hand (sometimes called crowdsourcing [40]). In some areas of physics, this technique has been used since the 1990s and is considered the only way results can be trusted [38,40]. We have not heard of any attempt of using blind analysis or crowdsourcing in ecology or evolution, but there are examples of networks of researchers collecting data under standardized methods, which sometimes is also called crowdsourcing [41,42].

### 2.3. Practical barriers

#### 2.3.1. The fallacy of factorial design

The use of multiple hypotheses also creates practical barriers, those that limit our ability and motivation to seriously consider multiple alternative hypotheses in our scientific investigations. One of these barriers is practical limitations inherent to factorial design, the standard experimental design that allows researchers to evaluate several explanatory variables and their interactions in the same study, one variable at a time [43]. Although the use of factorial design is very effective in testing alternative predictions, it often requires an inordinately high number of trials if one wants to seriously study patterns and potential interactions that might differ across multiple spatial and temporal scales. Every new hypothesis represents at least one more treatment or observational level that needs incorporation into the study design, hence adding treatments to a factorial design inevitably leads to geometrical increase in replicates, which by their very nature are often both time consuming and costly. We call this barrier the *fallacy of factorial design*, because, although conceptually easy to understand and clearly effective, complex factorial designs can impose serious practical challenges that may be insurmountable; complex factorial designs are simply not a practical option in evaluating many ecological problems.

A sobering example of the *fallacy of factorial design* is provided by Wilbur [44]. He and his collaborators had created an elegant system of both experimental and natural ponds to test several alternative hypotheses about how trophic connections among taxa in ecological communities are regulated. In one of his experiments, he used 16 treatments replicated four times in 64 tanks to study how pairwise interactions among species influence the dynamical outcome of multispecies assemblages. For sound procedural reasons, Wilbur used the same initial density for all species. As Wilbur pointed out, initial density could have an effect on the results. To properly extend the basic factorial design to test this single additional assertion at three initial densities would require a 3 × 3 × 3 × 3 factorial design (i.e. 81 treatments), leading to an insurmountable requirement for 324 ponds even using a modest number of four replicates!

#### 2.3.2. Simplicity

Although we are attracted to complex problems, our minds prefer easy, simple answers. In science, we place much cognitive value on simplicity [45]. Thus, any study that has a simple, easy-to-understand explanation will be preferred over a study that employs complex and perhaps less-elegant ideas. As a result, we might find ourselves in trouble when trying to publish a study that does not support a single hypothesis. Manuscripts presenting a single hypothesis are potentially much easier to review than manuscripts with multiple hypotheses, simply owing to easier communication between authors and editors/reviewers. Multiple hypothesis testing is certainly difficult to conduct, but just as importantly it is also difficult to explain and understand. As a consequence, papers that employ multiple hypothesis testing might appear ambiguous or confusing.

#### 2.3.3. Publication bias

When discussing intellectual barriers, we emphasized how scientists, just like other people, tend to favour previous knowledge over simple logic (*belief bias*)*.* This cognitive bias also creates a practical barrier, because editors and reviewers tend to rely on prior knowledge when evaluating a manuscript, creating additional difficulties for researchers when publishing studies that confront well-established ideas. This tension between new and old ideas could reflect a conflict between new and old generations. According to the philosopher Thomas Kuhn, such paradigms are only likely to be replaced when an entire generation leaves the stage and makes room for new researchers embracing a different point of view [46]. On the other hand, there is evidence suggesting that the important contributions are likely to be made at any point of a researcher's career [47]. Nevertheless, prior beliefs held by editors and reviews, with methodological and conceptual complexity, may present a substantial barrier to publishing multiple hypothesis studies.

### 2.4. How can we overcome practical barriers?

The fallacy of factorial design can be minimized by defining an *a priori* set of reasonable multiple hypotheses and their resulting patterns, thus focusing on measuring only those aspects that should help tease apart the predictions that arise from these hypotheses [1,7]. We should pay particular attention to hypotheses that generate similar predictions. Here a combination of decision trees and mathematical models can augment experimental approaches to greatly improve our ability to evaluate multiple hypotheses. Decision trees are designed to identify context-dependent associations among multiple-correlated predictor variables. For example, consider evaluating the relationship between social rank (e.g. ‘dominant’ or ‘subordinate’) and an array of potential predictors (e.g. age, mass, coloration, the presence of certain alleles, steroid levels, etc.). A decision tree is a classifier constructed from a series of nested decision points, each providing a binary outcome (left or right branch) based on one of the predictors (e.g. if male, right branch, else, left branch; if age more than 2, right branch, else, left branch). The order of nestedness within the tree allows identification of critical interactions between predictors (if [age more than 2?] is the first branching under sex = male, but it is absent under sex = female, there is a strong interaction between sex and age, where age matters for males but not for females). Several studies suggest that decision trees are superior to traditional regression linear methods because they require fewer assumptions, such as independence among observations and specific underlying statistical distributions of variables [48,49]. Although decision trees have certainly been used in ecology and evolution [49], considering the complexity of ecological and evolutionary problems, we believe that the technique is underused.

Mathematical models are also important tools when considering multiple hypotheses because they can explicitly reveal the internal consistency of a hypothesis and potential outcomes [50]. Some of these outcomes could be first tested under laboratory conditions before spending large amounts of money and years in the field. Using mathematical models to inform laboratory experiments is a well-known practice in ecology and evolution [51–53], but their use to guide fieldwork is less common. We believe much could be gained with this practice. For example, in an elegant paper, Turchin and Hanski translated hypotheses to explain rodent population cycles into parametrized quantitative mathematical models and compared the models with data. They found that predation offered the most parsimonious explanation for these cycles and emphasized that the arguments against the other explanations should be used to guide experimental tests [54].

## 3. Conclusions

More than any other scientific field, ecological and evolutionary research is aimed at understanding patterns arising from nonlinear and stochastic interactions among a multitude of processes and agents at multiple spatial and temporal scales. If we wish to truly advance scientific progress in spite of this complexity, we must better commit to strong inference in our scientific inquiries by simultaneously evaluating multiple competing hypotheses. While these ideas are not new, and have been vocally promoted by the scientific community for many years, we have tried to better understand why their practical application is still very much lacking. In so doing, we aim to raise awareness of the many sides to this complex issue, in the hope that it may encourage more rapid scientific progress in ecological and evolutionary research.

## Supplementary Material

A classified list of surveyed papers

## Data accessibility

Data are available in the electronic supplementary material.
