# Supplementary material for: Why are we not evaluating multiple competing hypotheses in ecology and evolution?
Source: R Soc Open Sci. 2017 Jan 11;4(1):160756. doi: 10.1098/rsos.160756 (PMC5319344; doi:10.1098/rsos.160756)
Supplement: A classified list of surveyed papers [file rsos160756supp1.pdf]

Electronic Supplementary Information

Why are we not evaluating multiple competing hypotheses in ecology and evolution?

Gustavo S. Betini[1 *†], Tal Avgar[1,2†], John M. Fryxell[1]

[†]These authors contributed equally to the manuscript

[1] Department of Integrative Biology, University of Guelph, Guelph, Ontario N1G 2W1, Canada

[2] Department of Biological Sciences, University of Alberta, Edmonton T6G 2E9, Canada

[*]e-mail: gsbetini@gmail.com

A classified list of surveyed papers

*asterisk marks examination of multiple non-competing (i.e., pertaining to different patterns) hypotheses. Papers were randomly selected from each of five leading journals in the fields of ecology and evolution: Ecology, Ecology Letters, Molecular Ecology, Evolution, and Global Change Biology (see Methods for details). Our classification does not represent any judgment about the value and/or the quality of the papers.

Application Motivated Inquiries (AMI)

No hypotheses

1. Beringer, J., Hutley, L. B., Tapper, N. J. & Cernusak, L. A. 2007 Savanna fires and their impact on net ecosystem productivity in North Australia. *Glob. Chang. Biol.* **13**, 990–1004. (doi:10.1111/j.1365-2486.2007.01334.x)

2. Bradley, B. A., Houghton, R. A., Mustard, J. F. & Hamburg, S. P. 2006 Invasive grass reduces aboveground carbon stocks in shrublands of the Western US. *Glob. Chang. Biol.* **12**, 1815–1822. (doi:10.1111/j.1365-2486.2006.01232.x)

3. Cloern, J. E. & Jassby, A. D. 2008 Complex seasonal patterns of primary producers at the land-sea interface. *Ecol. Lett.* **11**, 1294–303. (doi:10.1111/j.1461-0248.2008.01244.x)

4. Fornara, D. a., Steinbeiss, S., McNAMARA, N. P., Gleixner, G., Oakley, S., Poulton, P. R., Macdonald, a. J. & Bardgett, R. D. 2011 Increases in soil organic carbon sequestration can reduce the global warming potential of long-term liming to permanent grassland. *Glob. Chang. Biol.* **17**, 1925–1934. (doi:10.1111/j.1365-2486.2010.02328.x)

5. Gantenbein, B. & Keightley, P. D. 2004 Rates of molecular evolution in nuclear genes of east Mediterranean scorpions. *Evolution.* **58**, 2486–97. (doi: http:// dx.doi.org/10.1554/04-463)

6. Haile, S. G., Nair, V. D. & Nair, P. K. R. 2010 Contribution of trees to carbon storage in soils of silvopastoral systems in Florida, USA. *Glob. Chang. Biol.* **16**, 427–438. (doi:10.1111/j.1365-2486.2009.01981.x)

7. Howard, E. A., Gower, S. T. & Foley, J. A. 2004 Effects of logging on carbon dynamics of a jack pine forest in Saskatchewan, Canada. *Glob. Chang. Biol.* **10**, 1267–1284. (doi:10.1111/j.1365-2486.2004.00804.x)

8. Lewison, R. L., Freeman, S. A. & Crowder, L. B. 2004 Quantifying the effects of fisheries on threatened species: the impact of pelagic longlines on loggerhead and leatherback sea turtles. *Ecol. Lett.* **7**, 221–231. (doi:10.1111/j.1461-0248.2004.00573.x)

9. Lu, F., Wang, X., Han, B., Ouyang, Z., Duan, X., Zheng, H. & Miao, H. 2009 Soil carbon sequestrations by nitrogen fertilizer application, straw return and no-tillage in China's cropland. *Glob. Chang. Biol.* **15**, 281–305. (doi:10.1111/j.1365-2486.2008.01743.x)

10. Lund, M. et al. 2009 Variability in exchange of CO_2 across 12 northern peatland and tundra sites. *Glob. Chang. Biol.* **16**, 2436–2448. (doi:10.1111/j.1365-2486.2009.02104.x)

11. Mavárez, J., Amarista, M., Pointier, J.-P. & Jarne, P. 2002 Fine-scale population structure and dispersal in *Biomphalaria glabrata*, the intermediate snail host of *Schistosoma mansoni*, in Venezuela. *Mol. Ecol.* **11**, 879–89. (doi:10.1046/j.1365-294X.2002.01486.x)

12. Palmroth, S., Maier, C. A., McCarthy, H. R., Oishi, A. C., Kim, H.-S., Johnsen, K. H., Katul, G. G. & Oren, R. 2005 Contrasting responses to drought of forest floor CO_2 efflux

in a Loblolly pine plantation and a nearby Oak-Hickory forest. *Glob. Chang. Biol.* **11**, 421–434. (doi:10.1111/j.1365-2486.2005.00915.x)

13. Rendell, S. & Ennos, R. A. 2003 Chloroplast DNA diversity of the dioecious European tree *Ilex aquifolium L.* (English holly). *Mol. Ecol.* **12**, 2681–2688. (doi:10.1046/j.1365-294X.2003.01934.x)
14. Vasseur, D. & Yodzis, P. 2004 The color of environmental noise. *Ecology* **85**, 1146–1152. (doi:10.1890/02-3122)
15. Zidana, H., Turner, G. F., Van Oosterhout, C. & Hänfling, B. 2009 Elevated mtDNA diversity in introduced populations of *Cynotilapia afra* (Günther 1894) in Lake Malawi National Park is evidence for multiple source populations and hybridization. *Mol. Ecol.* **18**, 4380–4389. (doi:10.1111/j.1365-294X.2009.04362.x)

One hypothesis

1. Campos-Krauer, J. M. & Wisely, S. M. 2011 Deforestation and cattle ranching drive rapid range expansion of capybara in the Gran Chaco ecosystem. *Glob. Chang. Biol.* **17**, 206–218. (doi:10.1111/j.1365-2486.2010.02193.x)
2. Wimp, G. M., Young, W. P., Woolbright, S. A., Martinsen, G. D., Keim, P. & Whitham, T. G. 2004 Conserving plant genetic diversity for dependent animal communities. *Ecol. Lett.* **7**, 776–780. (doi:10.1111/j.1461-0248.2004.00635.x)

One exhaustive hypothesis

1. Bensch, S., Irwin, D. E., Irwin, J. H., Kvist, L. & Akesson, S. 2006 Conflicting patterns of mitochondrial and nuclear DNA diversity in *Phylloscopus* warblers. *Mol. Ecol.* **15**, 161–171. (doi:10.1111/j.1365-294X.2005.02766.x)
2. Kerby, J. L., Richards-Hrdlicka, K. L., Storfer, A. & Skelly, D. K. 2010 An examination of amphibian sensitivity to environmental contaminants: are amphibians poor canaries? *Ecol. Lett.* **13**, 60–67. (doi:10.1111/j.1461-0248.2009.01399.x)

3. McSwiney, C. P. & Robertson, G. P. 2005 Nonlinear response of N_2O flux to incremental fertilizer addition in a continuous maize (*Zea mays L.*) cropping system. *Glob. Chang. Biol.* **11**, 1712–1719. (doi:10.1111/j.1365-2486.2005.01040.x)

Pattern Motivated Inquiries (PMI)

No hypotheses

1. Blanckenhorn, W. & Hellriegel, B. 2002 Against Bergmann's rule: fly sperm size increases with temperature. *Ecol. Lett.* **5**, 7–10. (doi:10.1046/j.1461-0248.2002.00298.x)
2. Christin, P.-A., Sage, T. L., Edwards, E. J., Ogburn, R. M., Khoshravesh, R. & Sage, R. F. 2011 Complex evolutionary transitions and the significance of C_3-C_4 intermediate forms of photosynthesis in *Molluginaceae*. *Evolution* **65**, 643–660. (doi:10.1111/j.1558-5646.2010.01168.x)
3. Moore, C. M., Mills, M. M., Milne, A., Langlois, R., Achterberg, E. P., Lochte, K., Geider, R. J. & La Roche, J. 2006 Iron limits primary productivity during spring bloom development in the central North Atlantic. *Glob. Chang. Biol.* **12**, 626–634. (doi:10.1111/j.1365-2486.2006.01122.x)
4. O'Brien, H. E., Miadlikowska, J. & Lutzoni, F. 2009 Assessing reproductive isolation in highly diverse communities of the lichen-forming fungal genus *peltigera*. *Evolution* **63**, 2076–2086. (doi:10.1111/j.1558-5646.2009.00685.x)
5. Yukilevich, R. & True, J. R. 2008 African morphology, behavior and pheromones underlie incipient sexual isolation between US and Caribbean *Drosophila melanogaster*. *Evolution* **62**, 2807–2828. (doi:10.1111/j.1558-5646.2008.00488.x)

One hypothesis

1. Agrawal, A. A., Conner, J. K. & Johnson, M. T. J. 2002 Ecological genetics of an induced plant defense against herbivores: additive genetic variance and costs of phenotypic plasticity. *Evolution* **56**, 2206-2213. (doi:10.1111/j.0014-3820.2002.tb00145.x)

2. Banke, S. & McDonald, B. A 2005 Migration patterns among global populations of the pathogenic fungus *Mycosphaerella graminicola*. *Mol. Ecol.* **14**, 1881–1896. (doi:10.1111/j.1365-294X.2005.02536.x)*

3. Benner, J. W. & Vitousek, P. M. 2007 Development of a diverse epiphyte community in response to phosphorus fertilization. *Ecol. Lett.* **10**, 628–636. (doi:10.1111/j.1461-0248.2007.01054.x)

4. Bracken, M. E. S., Gonzalez-Dorantes, C. A. & Stachowicz, J. J. 2007 Whole-community mutualism: associated invertebrates facilitate a dominant habitat-forming seaweed. *Ecology* **88**, 2211–2219. (doi:10.1890/06-0881.1)

5. Fry, C. L. & Wilkinson, G. S. 2004 Sperm survival in female stalk-eyed flies depends on seminal fluid and meiotic drive. *Evolution* **58**, 1622–1626. (doi:10.1111/j.0014-3820.2004.tb01743.x)

6. Goetz, F. et al. 2010 A genetic basis for the phenotypic differentiation between siscowet and lean lake trout (*Salvelinus namaycush*). *Mol. Ecol.* **19**, 176–196. (doi:10.1111/j.1365-294X.2009.04481.x)

7. Hairston, J. N., Kearns, C. & Demma, L. P. 2005 Species-specific Daphnia phenotypes: a history of industrial pollution and pelagic ecosystem response. *Ecology* **116**, 1669-1678. (doi:10.1890/03-0784)

8. Kudo, G. & Ida, T. 2008 Linkages between phenology, pollination, photosynthesis, and reproduction in deciduous forest understory plants. *Ecology* **89**, 321–331. (doi:10.1890/06-2131.1)

9. Piertney, S. B. et al. 2008 Temporal changes in kin structure through a population cycle in a territorial bird, the red grouse *Lagopus lagopus scoticus*. *Mol. Ecol.* **17**, 2544–2551. (doi:10.1111/j.1365-294X.2008.03778.x)

10. Rossetto, M., Crayn, D., Ford, A., Mellick, R. & Sommerville, K. 2009 The influence of environment and life-history traits on the distribution of genes and individuals: a comparative study of 11 rainforest trees. *Mol. Ecol.* **18**, 1422–1438. (doi:10.1111/j.1365-294X.2009.04111.x)*

11. Tiwari, M., Bjorndal, K. A., Bolten, A. B. & Bolker, B. M. 2005 Intraspecific application of the mid-domain effect model: spatial and temporal nest distributions of green turtles,

Chelonia mydas, at Tortuguero, Costa Rica. *Ecol. Lett.* **8**, 918–924. (doi:10.1111/j.1461-0248.2005.00793.x)

12. Verboom, G. & Linder, H. 2003 Phylogenetics of the grass genus *Ehrharta*: evidence for radiation in the summer-arid zone of the South African cape. *Evolution* **57**, 1008–1021. (doi:10.1111/j.0014-3820.2003.tb00312.x)
13. Wallenstein, M. D., Mcmahon, S. K. & Schimel, J. P. 2009 Seasonal variation in enzyme activities and temperature sensitivities in Arctic tundra soils. *Glob. Chang. Biol.* **15**, 1631–1639. (doi:10.1111/j.1365-2486.2008.01819.x)*
14. Wiens, J. J., Pyron, R. A. & Moen, D. S. 2011 Phylogenetic origins of local-scale diversity patterns and the causes of Amazonian megadiversity. *Ecol. Lett.* **14**, 643–652. (doi:10.1111/j.1461-0248.2011.01625.x)*
15. Wilson, K., Cotter, S. C., Reeson, A. F. & Pell, J. K. 2001 Melanism and disease resistance in insects. *Ecol. Lett.* **4**, 637–649. (doi:10.1046/j.1461-0248.2001.00279.x)

One exhaustive hypothesis

1. Bokhorst, S., Bjerke, J. W., Street, L. E., Callaghan, T. V. & Phoenix, G. K. 2011 Impacts of multiple extreme winter warming events on sub-Arctic heathland: phenology, reproduction, growth, and CO_2 flux responses. *Glob. Chang. Biol.* **17**, 2817–2830. (doi:10.1111/j.1365-2486.2011.02424.x)*
2. Breshears, D., McDowell, N. & Goddard, K. 2008 Foliar absorption of intercepted rainfall improves woody plant water status most during drought. *Ecology* **89**, 41–47. (doi:10.1890/07-0437.1)
3. Greenberg, R. & Olsen, B. 2010 Bill size and dimorphism in tidal-marsh sparrows: island-like processes in a continental habitat. *Ecology* **91**, 2428–36. (doi:10.1890/09-1136.1)
4. Rolian, C., Lieberman, D. E. & Hallgrímsson, B. 2009 The co-evolution of human hands and feet. *Evolution* **64**, 1558–1568. (doi:10.1111/j.1558-5646.2009.00944.x)
5. Shimizu, K. K., Shimizu-Inatsugi, R., Tsuchimatsu, T. & Purugganan, M. D. 2008 Independent origins of self-compatibility in *Arabidopsis thaliana*. *Mol. Ecol.* **17**, 704–
714. (doi:10.1111/j.1365-294X.2007.03605.x)

Two hypotheses

1. Audzijonyte, A., Daneliya, M. E. & Väinölä, R. 2006 Comparative phylogeography of Ponto-Caspian mysid crustaceans: isolation and exchange among dynamic inland sea basins. *Mol. Ecol.* **15**, 2969–2984. (doi:10.1111/j.1365-294X.2006.03018.x)
2. Bridle, J. R., Baird, S. J. & Butlin, R. K. 2001 Spatial structure and habitat variation in a grasshopper hybrid zone. *Evolution* **55**, 1832–43. (doi:10.1111/j.0014-3820.2001.tb00832.x)
3. Pautasso, M. & Gaston, K. J. 2005 Resources and global avian assemblage structure in forests. *Ecol. Lett.* **8**, 282–289. (doi:10.1111/j.1461-0248.2005.00724.x)
4. Scheffer, S. J. & Grissell, E. E. 2003 Tracing the geographical origin of *Megastigmus transvaalensis* (*Hymenoptera*: *Torymidae*): an African wasp feeding on a South American plant in North America. *Mol. Ecol.* **12**, 415–21. (doi:10.1046/j.1365-294X.2003.01725.x)
5. Vargas, R., Detto, M., Baldocchi, D. D. & Allen, M. F. 2010 Multiscale analysis of temporal variability of soil CO_2 production as influenced by weather and vegetation. *Glob. Chang. Biol.* **16**, 1589–1605. (doi:10.1111/j.1365-2486.2009.02111.x)

Two exhaustive hypotheses

1. Holmes, M. J., Oldroyd, B. P., Allsopp, M. H., Lim, J., Wossler, T. C. & Beekman, M. 2010 Maternity of emergency queens in the Cape honey bee, *Apis mellifera capensis*. *Mol. Ecol.* **19**, 2792–2799. (doi:10.1111/j.1365-294X.2010.04683.x)

More than two hypotheses

1. Carstens, B. C., Brunsfeld, S. J., Demboski, J. R., Good, J. M. & Sullivan, J. 2005 Investigating the evolutionary history of the Pacific Northwest mesic forest ecosystem: hypothesis testing within a comparative phylogeographic framework. *Evolution* **59**, 1639–1652. (doi:10.1111/j.0014-3820.2005.tb01815.x)

2. Fernández, M., Astorga, A., Navarrete, S. A., Valdovinos, C. & Marquet, P. A. 2009 Deconstructing latitudinal species richness patterns in the ocean: does larval development hold the clue? *Ecol. Lett.* **12**, 601–611. (doi:10.1111/j.1461-0248.2009.01315.x)

3. Hammond, J. & Luttbeg, B. 2007 Predator and prey space use: dragonflies and tadpoles in an interactive game. *Ecology* **88**, 1525–1534. (doi:10.1890/06-1236)

4. Masaki, T. & Nakashizuka, T. 2002 Seedling demography of *Swida controversa*: effect of light and distance to conspecifics. *Ecology* **83**, 3497–3507. (doi:10.1890/0012-9658(2002)083[3497:SDOSCE]2.0.CO;2)

5. Riginos, C. & Nachman, M. W. 2001 Population subdivision in marine environments: the contributions of biogeography, geographical distance and discontinuous habitat to genetic differentiation in a blennioid fish, *Axoclinus nigricaudus*. *Mol. Ecol.* **10**, 1439–1453. (doi:10.1046/j.1365-294X.2001.01294.x)

Theory Motivated Inquiries (TMI)

No hypotheses

1. Brose, U., Jonsson, T., Berlow, E. & Warren, P. 2006 Consumer-resource body-size relationships in natural food webs. *Ecology* **87**, 2411–2417. (doi:10.1890/0012-9658(2006)87[2411:CBRINF]2.0.CO;2)

2. Feener, D. H., Orr, M. R., Wackford, K. M., Longo, J. M., Benson, W. W. & Gilbert, L. E. 2008 Geographic variation in resource dominance-discovery in Brazilian ant communities. *Ecology* **89**, 1824–1836. (doi:10.1890/07-0659.1)

3. Marrs, R. A, Sforza, R. & Hufbauer, R. A. 2008 Evidence for multiple introductions of *Centaurea stoebe micranthos* (spotted knapweed, *Asteraceae*) to North America. *Mol. Ecol.* **17**, 4197–4208. (doi:10.1111/j.1365-294X.2008.03903.x)

4. Murray, T. R., Frank, D. A. & Gehring, C. A. 2010 Ungulate and topographic control of arbuscular mycorrhizal fungal spore community composition in a temperate grassland. *Ecology* **91**, 815–827. (doi:10.1890/09-0209.1)

One hypothesis

1. Agrawal, A. A. & Kotanen, P. M. 2003 Herbivores and the success of exotic plants: a phylogenetically controlled experiment. *Ecol. Lett.* **6**, 712–715. (doi:10.1046/j.1461-0248.2003.00498.x)
2. Allan, E., van Ruijven, J. & Crawley, M. J. 2010 Foliar fungal pathogens and grassland biodiversity. *Ecology* **91**, 2572–2582. (doi:10.1890/09-0859.1)*
3. Altermatt, F. & Ebert, D. 2008 Genetic diversity of *Daphnia magna* populations enhances resistance to parasites. *Ecol. Lett.* **11**, 918–928. (doi:10.1111/j.1461-0248.2008.01203.x)
4. Averill, C. & Finzi, A. 2011 Increasing plant use of organic nitrogen with elevation is reflected in nitrogen uptake rates and ecosystem δ^{15}N. *Ecology* **92**, 883–891. (doi:0.1890/10-0746.1)
5. Chek, A. A., Bogart, J. P. & Lougheed, S. C. 2003 Mating signal partitioning in multi-species assemblages: a null model test using frogs. *Ecol. Lett.* **6**, 235–247. (doi:10.1046/j.1461-0248.2003.00420.x)*
6. Collin, R. 2006 Sex ratio, life-history invariants, and patterns of sex change in a family of protandrous gastropods. *Evolution* **60**, 735–745. (doi:10.1111/j.0014-3820.2006.tb01152.x)
7. Craig, J. K., Burke, B. J., Crowder, L. B. & Rice, J. A. 2006 Prey growth and size-dependent predation in juvenile estuarine fishes: experimental and model analyses. *Ecology* **87**, 2366–2377. (doi:10.1890/0012-9658(2006)87[2366:PGASPI]2.0.CO;2)
8. Douglass, J. G., Duffy, J. E. & Bruno, J. F. 2008 Herbivore and predator diversity interactively affect ecosystem properties in an experimental marine community. *Ecol. Lett.* **11**, 598–608. (doi:10.1111/j.1461-0248.2008.01175.x)*
9. Dutech, C., Maggia, L., Tardy, C., Joly, H. I. & Jarne, P. 2003 Tracking a genetic signal of extinction-recolonization events in a neotropical tree species: *Vouacapoua americana* Aublet in French Guiana. *Evolution* **57**, 2753–2764. (doi:10.1111/j.0014-3820.2003.tb01517.x)
10. Fukushima, M. 2001 Salmonid habitat-geomorphology relationships in low-gradient streams. *Ecology* **82**, 1238–1246. (doi:10.1890/0012-9658(2001)082[1238:SHGRIL]2.0.CO;2)

11. Housman, D. C., Zitzer, S. F., Huxman, T. E. & Smith, S. D. 2003 Functional ecology of shrub seedlings after a natural recruitment event at the Nevada Desert FACE Facility. *Glob. Chang. Biol.* **9**, 718–728. (doi:10.1046/j.1365-2486.2003.00618.x)

12. Karowe, D. N. 2007 Are legume-feeding herbivores buffered against direct effects of elevated carbon dioxide on host plants? A test with the sulfur butterfly, *Colias philodice*. *Glob. Chang. Biol.* **13**, 2045–2051. (doi:10.1111/j.1365-2486.2007.01422.x)

13. Matocq, M. D. 2004 Reproductive success and effective population size in woodrats (*Neotoma macrotis*). *Mol. Ecol.* **13**, 1635–1642. (doi:10.1111/j.1365-294X.2004.02173.x)

14. Mikolajewski, D. J., De Block, M., Rolff, J., Johansson, F., Beckerman, A. P. & Stoks, R. 2010 Predator-driven trait diversification in a dragonfly genus: covariation in behavioral and morphological antipredator defense. *Evolution* **64**, 3327–3325. (doi:10.1111/j.1558-5646.2010.01078.x)

15. Mills, S. C., Alatalo, R. V, Koskela, E., Mappes, J., Mappes, T. & Oksanen, T. A. 2007 Signal reliability compromised by genotype-by-environment interaction and potential mechanisms for its preservation. *Evolution* **61**, 1748–1757. (doi:10.1111/j.1558-5646.2007.00145.x)

16. Paoli, G. & Curran, L. 2005 Phosphorus efficiency of Bornean rain forest productivity: evidence against the unimodal efficiency hypothesis. *Ecology* **86**, 1548-1561. (doi:10.1890/04-1126)

17. Parzer, H. F. & Moczek, A. P. 2008 Rapid antagonistic coevolution between primary and secondary sexual characters in horned beetles. *Evolution* **62**, 2423–2428. (doi:10.1111/j.1558-5646.2008.00448.x)

18. Radwan, J., Kawałko, A., Wójcik, J. M. & Babik, W. 2007 MHC-DRB3 variation in a free-living population of the European bison, *Bison bonasus*. *Mol. Ecol.* **16**, 531–540. (doi:10.1111/j.1365-294X.2006.03179.x)

19. Rudgers, J. A., Koslow, J. M. & Clay, K. 2004 Endophytic fungi alter relationships between diversity and ecosystem properties. *Ecol. Lett.* **7**, 42–51. (doi:10.1046/j.1461-0248.2003.00543.x)

20. Russo, S. E., Wiser, S. K. & Coomes, D. A. 2007 Growth-size scaling relationships of woody plant species differ from predictions of the Metabolic Ecology Model. *Ecol. Lett.* **10**, 889–901. (doi:10.1111/j.1461-0248.2007.01079.x)

21. Schütte, U. M. E., Abdo, Z., Foster, J., Ravel, J., Bunge, J., Solheim, B. & Forney, L. J. 2010 Bacterial diversity in a glacier foreland of the high Arctic. *Mol. Ecol.* **19**, 54–66. (doi:10.1111/j.1365-294X.2009.04479.x)

22. Warkentin, K., Currie, C. & Rehner, S. 2001 Egg-killing fungus induces early hatching of red-eyed treefrog eggs. *Ecology* **82**, 2860–2869. (doi:10.1890/0012-9658(2001)082[2860:EKFIEH]2.0.CO;2)

One exhaustive hypothesis

1. Kümmerli, R., Gardner, A., West, S. A. & Griffin, A. S. 2009 Limited dispersal, budding dispersal, and cooperation: an experimental study. *Evolution* **63**, 939–949. (doi:10.1111/j.1558-5646.2008.00548.x)

2. Newton, P. & Carran, R. 2003 Reduced water repellency of a grassland soil under elevated atmospheric CO_2. *Glob. Chang. Biol.* **10**, 1–4. (doi:10.1046/j.1529-8817.2003.00715.x)

Two hypotheses

1. Bonduriansky, R. & Rowe, L. 2005 Sexual selection, genetic architecture, and the condition dependence of body shape in the sexually dimorphic fly *Prochyliza xanthostoma* (*Piophilidae*). *Evolution.* **59**, 138–151. (doi:10.1111/j.0014-3820.2005.tb00901.x)

2. Cook-Patton, S. C., McArt, S. H., Parachnowitsch, A. L., Thaler, J. S. & Agrawal, A. A. 2011 A direct comparison of the consequences of plant genotypic and species diversity on communities and ecosystem function. *Ecology* **92**, 915–923. (doi:10.1890/10-0999.1)

3. Ficetola, G. F., Garner, T. W. J. & De Bernardi, F. 2007 Genetic diversity, but not hatching success, is jointly affected by postglacial colonization and isolation in the threatened frog, *Rana latastei*. *Mol. Ecol.* **16**, 1787–1797. (doi:10.1111/j.1365-294X.2006.03198.x)

4. Moon, D. C. & Stiling, P. 2004 The influence of a salinity and nutrient gradient on coastal vs. upland tritrophic complexes. *Ecology* **85**, 2709–2716. (doi:10.1890/03-4099)

5. Norden, N., Chazdon, R. L., Chao, A., Jiang, Y.-H. & Vílchez-Alvarado, B. 2009 Resilience of tropical rain forests: tree community reassembly in secondary forests. *Ecol. Lett.* **12**, 385–394. (doi:10.1111/j.1461-0248.2009.01292.x)

6. Olano-Marin, J., Mueller, J. C. & Kempenaers, B. 2011 Heterozygosity and survival in blue tits (*Cyanistes caeruleus*): contrasting effects of presumably functional and neutral loci. *Mol. Ecol.* **20**, 4028–4041. (doi:10.1111/j.1365-294X.2011.05177.x)

7. Roff, D. A. 2002 Inbreeding depression: tests of the overdominance and partial dominance hypotheses. *Evolution* **56**, 768–775. (doi:10.1111/j.0014-3820.2002.tb01387.x)

8. Rundle, H. D., Chenoweth, S. F. & Blows, M. W. 2006 The roles of natural and sexual selection during adaptation to a novel environment. *Evolution* **60**, 2218–2225. (doi:10.1554/06-249.1)

9. Tjoelker, M. G., Oleksyn, J., Reich, P. B. & Żytkowiak, R. 2008 Coupling of respiration, nitrogen, and sugars underlies convergent temperature acclimation in *Pinus banksiana* across wide-ranging sites and populations. *Glob. Chang. Biol.* **14**, 782–797. (doi:10.1111/j.1365-2486.2008.01548.x)

Two exhaustive hypotheses

1. Hua, X. & Wiens, J. J. 2010 Latitudinal variation in speciation mechanisms in frogs. *Evolution* **64**, 429–443. (doi:10.1111/j.1558-5646.2009.00836.x)

More than two hypotheses

1. Dovčiak, M. & Halpern, C. 2010 Positive diversity–stability relationships in forest herb populations during four decades of community assembly. *Ecol. Lett.* **13**, 1300–1309. (doi:10.1111/j.1461-0248.2010.01524.x)

2. Mitchell, C. & Tilman, D. 2002 Effects of grassland plant species diversity, abundance, and composition on foliar fungal disease. *Ecology* **83**, 1713–1726. (doi:10.1890/0012-9658(2002)083[1713:EOGPSD]2.0.CO;2)

3. Raveh, A., Kotler, B. P., Abramsky, Z. & Krasnov, B. R. 2011 Driven to distraction: detecting the hidden costs of flea parasitism through foraging behaviour in gerbils. *Ecol. Lett.* **14**, 47–51. (doi:10.1111/j.1461-0248.2010.01549.x)

Descriptive Inquiries

1. Dick, C. W., Roubik, D. W., Gruber, K. F. & Bermingham, E. 2004 Long-distance gene flow and cross-Andean dispersal of lowland rainforest bees (*Apidae: Euglossini*) revealed by comparative mitochondrial DNA phylogeography. *Mol. Ecol.* **13**, 3775–3785. (doi:10.1111/j.1365-294X.2004.02374.x)
2. Dickey, M.-H., Gauthier, G. & Cadieux, M.-C. 2008 Climatic effects on the breeding phenology and reproductive success of an arctic-nesting goose species. *Glob. Chang. Biol.* **14**, 1973–1985. (doi:10.1111/j.1365-2486.2008.01622.x)
3. Sorvari, S., Korhola, A. & Tompson, R. 2002 Lake diatom response to recent Arctic warming in Finnish Lapland. *Glob. Chang. Biol.* **8**, 171–181. (doi:10.1046/j.1365-2486.2002.00463.x)

R code to calculate the 95% confidence intervals from the literature review.

Values are provided in Figure 1 and Table 1 in the main text

```
#PMI = pattern-motivated inquiry
#TMI = theory-motivated inquiry
#AMI = application-motivated inquiry

#for the total
PMI<-c(rep(0,100-36),rep(1,36))
TMI<-c(rep(0,100-41),rep(1,41))
AMI<-c(rep(0,100-20),rep(1,20))
results.PMI<-array(NA,c(100000,1))
results.TMI<-results.PMI
results.AMI<-results.PMI
for (i in 1:nrow(results.PMI)){
  results.PMI[i]<-sum(sample(PMI,size=length(PMI), replace=TRUE))
  results.TMI[i]<-sum(sample(TMI,size=length(TMI), replace=TRUE))
  results.AMI[i]<-sum(sample(AMI,size=length(AMI), replace=TRUE))
}
```

```
quantile(results.PMI, probs=c(0.025,0.975))
quantile(results.TMI, probs=c(0.025,0.975))
quantile(results.AMI, probs=c(0.025,0.975))


#for those with more than 1
PMI<-c(rep(0,36-(10+6)),rep(1,10))
TMI<-c(rep(0,41-(12+3)),rep(1,12))
AMI<-c(rep(0,20-(0+3)),rep(1,0))
results.PMI<-array(NA,c(100000,1))
results.TMI<-results.PMI
results.AMI<-results.PMI
for (i in 1:nrow(results.PMI)){
 results.PMI[i]<-sum(sample(PMI,size=length(PMI), replace=TRUE))
 results.TMI[i]<-sum(sample(TMI,size=length(TMI), replace=TRUE))
 results.AMI[i]<-sum(sample(AMI,size=length(AMI), replace=TRUE))
}

quantile(results.PMI, probs=c(0.025,0.975))
quantile(results.TMI, probs=c(0.025,0.975))
quantile(results.AMI, probs=c(0.025,0.975))


#for those with exhaustive 1
PMI<-c(rep(0,36-(10+6)),rep(1,6))
TMI<-c(rep(0,41-(12+3)),rep(1,3))
AMI<-c(rep(0,20-(0+3)),rep(1,3))
results.PMI<-array(NA,c(100000,1))
results.TMI<-results.PMI
results.AMI<-results.PMI
for (i in 1:nrow(results.PMI)){
 results.PMI[i]<-sum(sample(PMI,size=length(PMI), replace=TRUE))
 results.TMI[i]<-sum(sample(TMI,size=length(TMI), replace=TRUE))
 results.AMI[i]<-sum(sample(AMI,size=length(AMI), replace=TRUE))
}

quantile(results.PMI, probs=c(0.025,0.975))
quantile(results.TMI, probs=c(0.025,0.975))
quantile(results.AMI, probs=c(0.025,0.975))
```
